# On the Cooling Treatment of Gout

**Published:** 1815-07

**Authors:** Robert Kinglake

**Affiliations:** Taunton


*For the Medical and Physical Journal.*

### *On the Cooling Treatment of Gout;* by Dr. Kinglake.

AFTER having already said so much on this subject, and after having furnished the public with ample data on which to reason and to conclude, it appeared to me to be proper and respectful to observe a patient silence, to allow the question to work its own course, to rest its procedure exclusively on facts, and to await the final issue of that testimony.

Nearly eight years have now elapsed since the public attention was last drawn by me to the subject. At that time much had been accomplished, and my opinion of the validity of the principle that had been asserted, and of the utility of the practice proposed, savoured, in the estimation of some persons, not a little of presumption, and was, in some instances, actually treated with unsparing harshness of censure. All this, and much more, it was my duty to endure, in what appeared to me to be the cause of an important truth. Bad reasoning and low calumny were opposed to my pretensions, which have been fully exposed and answered in a work entitled "Reviewers Reviewed," that has been fit for the press these seven years, and which, if the practical interests of the subject should still require its publication, will not be much longer withheld. In the mean time, it becomes me to state to the public, in a summary way, what has been the amount of my late experience on the cooling treatment of gout, with such other observations as may be judged relevant to the subject.

It will not be denied that my additional experience in this inquiry, during upwards of seven years, must have been sufficient either further to confirm or to shake my previous opinions. It will not be necessary for me to cite cases in all the personal and peculiar circumstances in which they have since presented. Enough of this sort of testimony has

 been been already adduced in my two volumes published on the treatment of gout.* I shall, therefore, on this occasion, merely state, that, amidst all the varieties of cases of gout that have for some years fallen under my notice, no instance has occurred to alter my firm persuasion of the superior and indeed adequate efficacy of the cooling treatment of that disease. On no occasion has the general health suffered in consequence of this practice; but in numerous instances it has been manifestly preserved and improved by its salutary influence. Not a suspicion even of apoplexy, palsy, dropsy, carbuncles, and mutilated senses and limbs, has happened, in a solitary instance, to countenance the absurd prognostics uttered against the practice by pseudo-critics, by the prejudiced, by the timid, by the garrulous, and by the vulgar. Not one of the killing and life enduring mischiefs thus hastily predicted, has been realised, to my knowledge.

If the experience of others has been different, the public should be made acquainted with it. The prurient disposition shown to disparage and cry down the practice, would have been *lynx-eyed* in espying grounds for impeaching it, but it has not been lately assailed: it is, therefore, just to infer, that it could not; and in this persuasion I shall rest satisfied that the practice has and will maintain its full claims to public adoption and confidence, and shall ever challenge, or rather intreat, those who may think differently, those who would willingly controvert the principles on which it is founded, or would refute the facts afforded by manifold experience, to do so, at any time, in a way that may merit attention, and they may be assured of every consideration that may be due to the occasion.

Popular prejudices cannot, under any circumstances, at once and entirely give way: they are so incorporated with the established habits and modes of thinking, as to form an integrant part of the understanding. It is no valid objection to a prejudice, in the opinion of its advocates, that it is not consonant with reason nor consistent with experience. Such an objection is met by an unqualified refusal to be convinced, by an avowed determination to be governed by accredited usage and the sanction of public belief. This is too much the propensity and conduct of the unthinking part of mankind, and the intellectual indifference on which it is founded is apt to be countenanced and upheld by the crafty and designing, who may have a personal interest in prolonging erroneous prejudice. This has been the case with

* See Dissertation on Gout, by Dr. Kinglake, 1804.—Additional Cases of Gout, &c. &c. 1807.

regard regard to the cooling treatment of gout; but the difficulty of carrying on the imposture in the very face of continually occurring proof of its pre-eminent advantages, has, in a great degree, already divested the public mind of these gratuitous terrors, by which the old system has been maintained and vindicated, against the evidence of superior benefit from the cooling mode of managing the disease.

In an inquiry like that of the treatment of gout, in which but a small portion of the community has a direct interest, it is difficult to induce that general conviction in favour of an altered practice which its importance may demand, and which individual experience upon a larger scale would be certain to insure it, yet the lapse of time that has occurred since its introduction, has done much towards finally subduing every prejudice against a free and adequate employ of the cooling treatment. The main part of difficulty in which the enemies of the cooling practice conceived they had firmly lodged themselves, is either voluntarily relinquished, or carried by innovators, whom they themselves have patronised. The *eau medicinale*, and other kindred nostrums, have been received by the advocates of the remedial and salutary character of gout as entitled to trial; and, although the leading feature of their medicinal influence is brisk evacuation, and, of course, a consequent reduction of strength, arising from that debilitating effect, yet no objection has been taken against them on that account. The idea of this evacuation removing noxious, that is to say the *peccant*, substances of the humeral pathologist, has probably reconciled this effect to the adopted prejudice of additional support and energy being necessary to reject the morbid cause of the affection. The *eau medicinale* has been of essential service in bending the public opinion to an acquiescence in the obvious benefit accruing to gouty irritation from briskly evacuating the first passages. What, indeed, can be more anti-inflammant and refrigerating than dislodging oppressive and stimulating substances from the alimentary canal, which, in fact, could not be retained without occasioning incalculable mischief during the existence of arthritic disease?

But how does this admitted relief quadrate with brandy, with flannel, and with heated and unventilated apartments? The contrast of quality and effect is well adapted to show the incongruousness of attempting to reconcile them to any common principle of action. All remedies, of whatever name or quality, that may from time to time be proposed for the cure of gout, will necessarily have but an ephemeral reputation, if they be not directed to the removal of the conditions ditions of vital action that constitute the disease. Various states often enter into the combination of disease. Simplicity cannot be expected where different powers and agencies are requisite to produce a given effect. The phenomena of life, as well as of disease are of this complicated nature, and can only be affected by causes capable of producing specific effects. The laws of sympathy and association in animal life admit of a wide scope of medicinal influence in a manner that would not appear to be attainable by the means employed; but in diseases of an inflammatory description, accompanied with severe pain, and extending its hurtful effects to the system at large, the mode in which relief should be attempted does not remain an object of uncertain speculation: it is sufficiently evident that the inflammatory action, with its essential conditions *heat* and *pain*, should be directly and as rapidly as possible overcome, when the morbid effects would necessarily cease. The only practical consideration, then, is, what would be at once the most efficient and salutary means of accomplishing the object. To this end, the use of topical cold to the affected part, abstinence, lax bowels, the avoidance of heated rooms, and free admission of cool air, have been proposed, and have proved equal to the desired effect; and thus the treatment has been justified by its fruits, and will, no doubt, be enabled at all times to show a fearless front against all speculatists, declaimers, calumniators, and false critics, however assailed and opposed.

Were it not that ample testimony has been already published, in the works before referred to, on the decisive efficacy and safety of the cooling treatment of gout, the stock of proof that has been amassed might be considerably extended by written communications in my possession, leaving me at full liberty to use them in whatever way may be judged most conducive to promote and establish the practice. It is my intention hereafter to select any thing of novelty that might occur, in which any question of importance on the subject might be fairly introduced and discussed, for the purpose of rendering the doctrine as unexceptionable in principle as it seems to be beneficial in practice.

If the experience of others could assist me in this proposed inquiry, it would be gratifying to me to receive it. My sole object is to elicit the truth, and to give it the publicity that it may appear to merit. It might be justly imputed to me as a dereliction of a duty to humanity, were I to withhold any unfavourable circumstance from public notice, in a research so interesting to mankind as that which relates to the most salutary treatment of gout. My not being able to state any circumstances at all disadvantageous to the cooling treatment of that disease, warrants me in concluding, that it is of that class of natural remedies that must survive all prejudices and cavillings against it, and that will ultimately accomplish its own establishment.

*Taunton;* ROBERT KINGLAKE.

*May* 1, 1815.

